# Supplementary material for: Screening of candidate substrates and coupling ions of transporters by thermostability shift assays
Source: eLife. 2018 Oct 15;7:e38821. doi: 10.7554/eLife.38821 (PMC6211832; doi:10.7554/eLife.38821)
Supplement: Supplementary file 1. [file elife-38821-supp1.docx]

| **Genus** | **Species** | **Transporter**  **proteins** | **Proteins with no predicted substrate** | **Percentage** |
| --- | --- | --- | --- | --- |
| *Acinetobacter* | *baumannii* | 391 | 75 | 19% |
| *Arabidopsis* | *thaliana* | 1279 | 311 | 24% |
| *Caenorhabditis* | *elegans* | 669 | 179 | 27% |
| *Campylobacter* | *-* | 197 | 37 | 19% |
| *Drosophila* | *melanogaster* | 662 | 105 | 16% |
| *Enterococcus* | *faecium* | 392 | 44 | 11% |
| *Escherichia* | *coli* | 638 | 120 | 19% |
| *Halobacterium* | *-* | 208 | 10 | 5% |
| *Helicobacter* | *pylori* | 140 | 33 | 24% |
| *Homo* | *sapiens* | 1472 | 102 | 7% |
| *Klebsiella* | *pneumoniae* | 879 | 164 | 19% |
| *Neisseria* | *gonorrhoeae* | 172 | 32 | 19% |
| *Oryza* | *sativa* | 1285 | 165 | 13% |
| *Pseudomonas* | *aeruginosa* | 703 | 147 | 21% |
| *Saccharomyces* | *cerevisiae* | 343 | 126 | 37% |
| *Salmonella* | *-* | 605 | 113 | 19% |
| *Shigella* | *-* | 534 | 95 | 18% |
| *Staphylococcus* | *aureus* | 348 | 41 | 12% |
| *Streptococcus* | *pneumoniae* | 307 | 50 | 16% |
| *Sulfolobus* | *-* | 205 | 18 | 9% |
| *Vibrio* | *cholerae* | 458 | 82 | 18% |
|  | Total | 11886 | 2048 | 18% |
