# Supplementary material for: Screening of candidate substrates and coupling ions of transporters by thermostability shift assays
Source: eLife. 2018 Oct 15;7:e38821. doi: 10.7554/eLife.38821 (PMC6211832; doi:10.7554/eLife.38821)
Supplement: Peer review history

# Decision letter

**DOI:** 10.7554/eLife.38821.020

**Affiliations:** J.W. Goethe-UniversityGermany

In the interests of transparency, eLife includes the editorial decision letter and accompanying author responses. A lightly edited version of the letter sent to the authors after peer review is shown, indicating the most substantive concerns; minor comments are not usually included.

Thank you for submitting your article "Screening of candidate substrates and coupling ions of transporters by thermostability shift assays" for consideration by *eLife*. Your article has been reviewed by three peer reviewers including Volker Dötsch as the Reviewing Editor and Reviewer #1, and the evaluation has been overseen by Richard Aldrich as the Senior Editor. The following individual involved in review of your submission has agreed to reveal their identity: Dirk Slotboom (Reviewer #3).

The reviewers have discussed the reviews with one another and the Reviewing Editor has drafted this decision to help you prepare a revised submission. Given that we consider this work to be more of a technical than a conceptual advance, we feel the contribution would be better placed in the category of a Tools and Resources paper than a Research Article.

Summary:

The substrates of many transporters, including those belonging to the human SLC family of transporters are unknown. In vitro transport assays are the gold standard, but for technical reasons these can be hard to establish. The Kunji lab nicely demonstrates how thermal shift assays with the CPM dye can be used in a high-throughput format to screen for potential substrates. The premise is simple. Only interacting ligands that show increased (high) thermostabilization are potential substrates. They show the usefulness of the CPM assay to identify the correct substrates and ligands for three different transport proteins and also show how it is able to also pick-up ion dependent (Na^+^) or (H^+^) substrate binding. They further show the use of the assay to identify potential substrates for an unknown mitochondrial carrier.

Essential revisions:

1) The de-orphanizing of the mitochondrial carrier is a very important result of this study and should be confirmed by a classical transport assay to validate the main conclusion that this is a phosphate transporter. Do other negatively charged anions, such as sulphate, show a change in its melting temperature?

2) The discussion of the data should be expanded. For instance, spermidine and spermine destabilize the phosphate transporter, but have no effect on the ADP/ATP carrier (AAC). Such observations should be discussed. Similarly, for the stabilizing effect of zinc chloride on AAC. Unfortunately, all substrates shown in Figure 2—figure supplement 4 were also tested for the phosphate transporter (Figure 3), apart from zinc. Why? Zinc ions seem interesting.

3) Related to this question, the authors claim: "Transported substrates bind only transiently and relatively weakly, leading to conformational change that in turn causes the release…" This is not strictly true. In some cases, transported substrate bind very tightly (aspartate to GltPh for instance). In addition, in detergent solution transporters are simply binding proteins that are characterized by an apparent K_d_, regardless of conformational changes. This part must be phrased more carefully.

4) What is the reliable working range of thermostabilization? In this setup it seems that Tm shifts as low as 2 degrees are significant. Is this empirically-based and/or are there recommendations based on other studies for using thermal shift assays for identifying potential ligands?

5) Some membrane proteins will be destabilised in DMSO. If the protein is too unstable in DMSO would this not make it difficult to measure a shift in thermostabilization? In other words, what is the working range of thermostability of the protein required for accurately measuring ligand binding by this method? Indeed, all of the transporters tested in this study had melting temperatures higher than 50 °C, which seem high and many membrane proteins, especially mammalian membrane proteins, will have lower melting temperatures.

6) Related, could this assay also be carried out with the membrane protein incorporated into nanodiscs or into bicelles obtained through detergent titration from nanodiscs (thus avoiding complications from MSP melting)? This would make it a useful assay for membrane proteins of non-thermophilic organisms, in particular mammalian proteins. Without this possibility this method would not change the current situation for mammalian transporters of guessing the ligand based on homology to bacterial transporters. Since the authors use human and eukaryotic transporters as justification for their method development in the Introduction, this issue should at least be discussed.

7) Data representations and statistics should be improved. For instance, in Figure 1—figure supplement 1, the authors state that data represent averages and SDs of 3 biological replicates. Yet, for the AMP data 4-5 data points, which is more than 3 were used, and there are no error bars with SD.

8) The explanation for the stabilizing effect of low pH (pH 6 compared to pH 8) on the phosphate transporter seems questionable (subsection “The effect of coupling ions**”**). Protonation of the glutamate residue in the binding site (pKa of 4) is less likely than protonation of the substrate pKa of 7.2.

9) The stock concentrations of ligands are very high (either 25 mM in buffer or 100 mM in DMSO). What was the final concentration actually used for library screening? Along these lines, how can one eliminate false positives due to non-specific interactions with such high concentrations of ligands? In particular hydrophobic compounds will easily become incorporated into the micelles and could cause effects.

# Reply: Author response

**DOI:** 10.7554/eLife.38821.021

Essential revisions:

1) The de-orphanizing of the mitochondrial carrier is a very important result of this study and should be confirmed by a classical transport assay to validate the main conclusion that this is a phosphate transporter. Do other negatively charged anions, such as sulphate, show a change in its melting temperature?

We have now demonstrated that the orphan mitochondrial carrier from *Tetrahymena thermophila* transports phosphate; we have included the data as (Figure 3—figure supplement 3) and we have submitted the data to the Dryad repository. We also tested the other compounds that gave a large shift in thermostability (glyoxylate, phosphoenolpyruvate and acetyl phosphate) for transport; none of these compounds are transported by the phosphate carrier. In competition assays, glyoxylate did inhibit phosphate uptake, showing that it can directly compete with phosphate transport when present in excess. We have included a brief discussion in the manuscript.

We have tested the effect of sulphate on the stability of both the mitochondrial phosphate carrier (Figure 3) and ADP/ATP carrier (Figure 2—figure supplement 4). There is a very small, but significant, stabilising effect of 0.3 °C for the phosphate carrier, but no statistically significant change in the melting temperature of the mitochondrial ADP/ATP carrier.

2) The discussion of the data should be expanded. For instance, spermidine and spermine destabilize the phosphate transporter, but have no effect on the ADP/ATP carrier (AAC). Such observations should be discussed. Similarly, for the stabilizing effect of zinc chloride on AAC. Unfortunately, all substrates shown in Figure 2—figure supplement 4 were also tested for the phosphate transporter (Figure 3), apart from zinc. Why? Zinc ions seem interesting.

Spermine and spermidine have a generic destabilising effect on all tested mitochondrial carrier family members (e.g. mitochondrial phosphate carrier, aspartate/glutamate carrier, YMC1 and ATP-Mg/Pi carriers) with the exception of the mitochondrial ADP/ATP carrier. Polyamines in general bind to surface charges of proteins and alter the solubility and stability of proteins (that is why they are additives in crystallisation screens). We have included a brief discussion in the manuscript.

We have also tested the effect of zinc on the phosphate carrier, but unfortunately the unfolding curves are uninterpretable and irreproducible (see Author response image 1), as we suspect that zinc addition leads to aggregation of the carrier. This is quite a special case, as we have not seen this effect with other metal ions.

**Author response image 1.**

3) Related to this question: the authors claim: "Transported substrates bind only transiently and relatively weakly, leading to conformational change that in turn causes the release…" This is not strictly true. In some cases, transported substrate bind very tightly (aspartate to GltPh for instance). In addition, in detergent solution transporters are simply binding proteins that are characterized by an apparent K_d_, regardless of conformational changes. This part must be phrased more carefully.

The purpose of this statement was to emphasise that substrates of transport proteins often have orders of magnitude lower binding affinities (K_d_ of ADP for AAC is ~10 μM) than inhibitors (K_d_ of CATR for AAC is ~10 nM), but the reviewer is right that this statement might not be universal. It is true that transporters in solution do not actually transport as there are no compartments. However, we have found that they do cycle through conformational changes in detergent micelles, similar to those observed in the membrane. Still, we have removed this sentence, as it is not pertinent to the contents of the manuscript.

4) What is the reliable working range of thermostabilization? In this setup it seems that T_m_ shifts as low as 2 degrees are significant. Is this empirically-based and/or are there recommendations based on other studies for using thermal shift assays for identifying potential ligands?

The working range of thermostabilisation assays varies widely, as it is protein-dependent and needs to be determined empirically. One of the advantages of this method is that the library contains compounds that will not bind and therefore will not stabilise the target protein, which can then act as internal controls, allowing even small positive results to be identified. We have added comments along these lines.

5) Some membrane proteins will be destabilised in DMSO. If the protein is too unstable in DMSO would this not make it difficult to measure a shift in thermostabilization? In other words, what is the working range of thermostability of the protein required for accurately measuring ligand binding by this method? Indeed, all of the transporters tested in this study had melting temperatures higher than 50 °C, which seem high and many membrane proteins, especially mammalian membrane proteins, will have lower melting temperatures.

Indeed, some membrane proteins might be destabilised in DMSO, but this would be immediately apparent from the absence of an unfolding curve. To avoid these complications, we prepare compound stocks in high concentrations (typically 100 mM as a 50-times stock in DMSO), therefore minimising the concentration of DMSO introduced into the assay. Control reactions can be carried out in the presence of the same amount of DMSO to verify the effect on protein stability. We have added a line to that effect in the Discussion.

The apparent melting temperature must not be confused with the half-life of a protein in detergent at that temperature. Mitochondrial carriers are among the most unstable eukaryotic membrane proteins with a half-life of less than 3h in dodecyl-maltoside buffers at 4 ^o^C (Bamber et al., 2006). They consist of three domains with few polar interactions between them and their transport mechanism involves six moving elements, making them the most dynamic transport proteins characterised to date. We have tested a range of other membrane proteins from different protein families and from different species (including mammalian and human membrane proteins heterologously expressed in *S. cerevisiae*) using our thermostability assay. We have found that the apparent melting temperatures of unliganded proteins typically varies from 40 – 55 °C. Temperature ramping using our program starts at 25 °C; in theory, as long as the protein is in a folded conformation at the start of the assay, unfolding curves can be generated with an apparent melting temperature as low as 30 °C. This is a broadly applicable method.

6) Related, could this assay also be carried out with the membrane protein incorporated into nanodiscs or into bicelles obtained through detergent titration from nanodiscs (thus avoiding complications from MSP melting)? This would make it a useful assay for membrane proteins of non-thermophilic organisms, in particular mammalian proteins. Without this possibility this method would not change the current situation for mammalian transporters of guessing the ligand based on homology to bacterial transporters. Since the authors use human and eukaryotic transporters as justification for their method development in the Introduction, this issue should at least be discussed.

We have successfully assayed proteins incorporated into both nanodiscs and amphipols. We have added a sentence to the text accordingly.

7) Data representations and statistics should be improved. For instance, in Figure 1—figure supplement 1, the authors state that data represent averages and SDs of 3 biological replicates. Yet, for the AMP data 4-5 data points, which is more than 3 were used, and there are no error bars with SD.

We apologise for this oversight, and have corrected the figure legend for Figure 1—figure supplement 1. We decided to plot each individual data point on this graph, rather than an average and standard deviation, which has now been changed.

8) The explanation for the stabilizing effect of low pH (pH 6 compared to pH 8) on the phosphate transporter seems questionable (subsection “The effect of coupling ions**”**). Protonation of the glutamate residue in the binding site (pKa of 4) is less likely than protonation of the substrate pKa of 7.2.

The point is that the proton binding site, a glutamate residue, and phosphate binding sites are located close together, as we have observed in many other proton-coupled mitochondrial carriers, such as the mitochondrial aspartate/glutamate carrier and citrate carrier. The premise is that the proton is shared between the negatively charged substrate and the carboxyl group of the glutamate residue, forming proton-mediated bonds that help to increase the interaction energy of phosphate binding. In support of this notion the transport rates are highest at pH 6 and virtually absent at pH 8 (Stappen and Kramer, 1994). We have added a better description and the appropriate reference to explain this better.

9) The stock concentrations of ligands are very high (either 25 mM in buffer or 100 mM in DMSO). What was the final concentration actually used for library screening? Along these lines, how can one eliminate false positives due to non-specific interactions with such high concentrations of ligands? In particular hydrophobic compounds will easily become incorporated into the micelles and could cause effects.

We have listed the stock concentrations of substrate in the Materials and methods. For clarity, we have added the final working concentrations of each library in the figure legends.

An advantage of this method is that generic stabilising and destabilising compounds will be identified as more proteins are tested. For example, spermine and spermidine destabilise the phosphate, aspartate/glutamate, ATP-Mg/Pi and YMC1 carriers. We have added a sentence in the Discussion.

References:

Bamber, L., Harding, M., Butler, P. J. G., and Kunji, E. R. S. (2006) Yeast mitochondrial ADP/ATP carriers are monomeric in detergents. *Proc. Natl. Acad. Sci. U.S.A.***103**, 16224-16229

Stappen, R., and Kramer, R. (1994) Kinetic mechanism of phosphate/phosphate and phosphate/OH- antiports catalyzed by reconstituted phosphate carrier from beef heart mitochondria. J. Biol. Chem. 269, 11240-11246
